# Amygdalin Attenuates Atherosclerosis and Plays an Anti-Inflammatory Role in ApoE Knock-Out Mice and Bone Marrow-Derived Macrophages

**DOI:** 10.3389/fphar.2020.590929

**Published:** 2020-10-29

**Authors:** Yiru Wang, Qingyun Jia, Yifan Zhang, Jing Wei, Ping Liu

**Affiliations:** ^1^ Longhua Hospital, Shanghai University of Traditional Chinese Medicine, Shanghai, China; ^2^ Second Ward of Trauma Surgery Department, Linyi People’s Hospital, Linyi, China; ^3^ Shanghai Xuhui Central Hospital, Shanghai, China

**Keywords:** atherosclerosis, inflammation, MAPKs, NF-κB p65, amygdalin, AP-1

## Abstract

Amygdalin, the main component of Prunus persica (L.) Stokes, has been used to treat atherosclerosis in mouse model due to its anti-inflammatory role. However, the underlying mechanism remains poorly understood. This study aimed to evidence the influence of amygdalin on high-fat diet-induced atherosclerosis in ApoE knock-out (ApoE^−/−^) mice, and unravel its anti-inflammatory mechanism. ApoE^−/−^ mice fed with high-fat diet for eight weeks were randomly divided into four groups and injected with amygdalin at the concentration of 0.08 or 0.04 mg/kg for 12 weeks. Additionally, bone marrow-derived macrophages were intervened with oxidized low-density lipoprotein (oxLDL) or lipopolysaccharide plus various concentrations of amygdalin for further exploration. Body weight, serum lipid profiles and inflammatory cytokines were detected by ELISA, gene expression by RT-PCR, plaque sizes by Oil Red O, lymphatic vessels of heart atrium and Tnfα production by immunofluorescence staining. MAPKs, AP-1 and NF-κB p65 pathways were also explored. Amygdalin decreased body weight, serum lipids, plaque size, lymphatic vessels and inflammatory cytokines (Il-6, Tnfα), Nos1 and Nos2, and increased Il-10 expression in ApoE^−/−^ mice. In oxLDL-induced bone marrow-derived macrophages, amygdalin reduced inflammatory cytokines (Il-6, Tnfα), Nos1 and Nos2, and increased Il-10 production. These effects were associated with the decreased phosphorylation of Mapk1, Mapk8, Mapk14, Fos and Jun, and the translocation of NF-κB p65 from nucleus to cytoplasm. The results suggested that amygdalin could attenuate atherosclerosis and play an anti-inflammatory role via MAPKs, AP-1 and NF-κB p65 signaling pathways in ApoE^−/−^ mice and oxLDL-treated bone marrow-derived macrophages.

## Introduction

Cardiovascular disease is a leading cause of death worldwide (Wachira and Stys 2013). Inflammation is a well-known risk factor underlying the pathogenesis of atherosclerosis, the common pathological basis of various cardiovascular and cerebrovascular diseases (Gao et al., 2017). The protective and destructive effects of inflammatory cascade are usually balanced (Patil et al., 2019). Whereas, chronic inflammation is usually characterized by substantial destruction and recovery of injured tissues from an inflammatory response (Nathan, 2002). If uncontrolled, inflammation may give rise to numerous diseases like rheumatoid arthritis, neurological disease, osteoarthritis, and coronary artery disease (CAD) (Fangkrathok et al., 2013; Ridker and Luscher 2014; Pelletier-Galarneau and Ruddy 2019; Woodell-May and Sommerfeld, 2020). Increased, enlarged and disordered lymphatic vessels in CAD patient’s heart have been previously reported (Kholova et al., 2011). Lymphatic vessels help transport the immune cells and regulate inflammatory responses. Abnormal lymphatic proliferation and remodeling may lead to continuous aggravation of various chronic inflammatory responses (Alitalo and Detmar, 2012). However, medicines treating atherosclerosis and inflammation are generally associated with untoward effects and low efficacy.

Activation of various immune cells, such as neutrophils, monocytes-macrophages and dendritic cells, contributes to atherosclerotic changes (Schaftenaar et al., 2016). Among these cell types, macrophages are the primary inflammatory cells involved in inflammatory progression (Jinnouchi et al., 2020; Kuznetsova et al., 2020). In early lesions, macrophages can clear away lipoprotein particles and apoptotic cells, and differentiate into foam cells that secrete pro-inflammatory molecules. However, in progressive lesions, macrophages are unable to engulf dead cells. Eventually, the necrotic core forms and expands with the accumulation of apoptotic debris. Necrotic core expansion is strongly associated with inflammation (Tabas, 2005). Macrophages usually derive from monocytes recruited in early-stage lesions, but still proliferate in the mouse with advanced atherosclerosis, once triggered by microenvironmental signals. Therefore, macrophages may be taken as the target in the treatment of atherosclerosis (Robbins et al., 2013).

In the case of hyperlipidemia, more monocyte-derived macrophages proliferate, differentiate into foam cells, and emigrate into atherosclerotic lesions, thus elevating the risk of atherosclerosis (Adamson and Leitinger, 2011). However, emerging evidence shows that hyperlipidemia not only activates monocytes but also induces neutrophilia and priming of circulating neutrophils. Hypercholesterolemia-induced neutrophilia is an initial stimulus for plaque development (Drechsler et al., 2010). The plaque harbors a minimal level of neutrophils (Rotzius et al., 2010), but these neutrophils can induce a large-scale infiltration of monocytes. Research has been conducted to understand the mechanisms of neutrophil–monocyte interactions in lesion formation (Ionita et al., 2010). One is the neutrophil-driven recruitment of monocytes to sites of lipid accumulation (Soehnlein et al., 2008). Additionally, the way the mature immune system reacts to inflammation is largely defined by the self-renewal and multilineage capacity of a rare population of hematopoietic stem and progenitor cells (HSPCs) in the bone marrow (Baldridge et al., 2011). In hypercholesterolemic ApoE^−/−^ and Ldlr^−/−^ mice, hyperlipidemia induces a substantial increase of HSPCs, and this myeloid skewing further induces monocytosis and granulocytosis, which aggravates atherosclerosis (Seijkens et al., 2014). Based on these, we speculate that macrophages are major players in atherosclerosis, and bone marrow-derived macrophages (BMDMs) might be a target in treating atherosclerosis.

**FIGURE 1:**
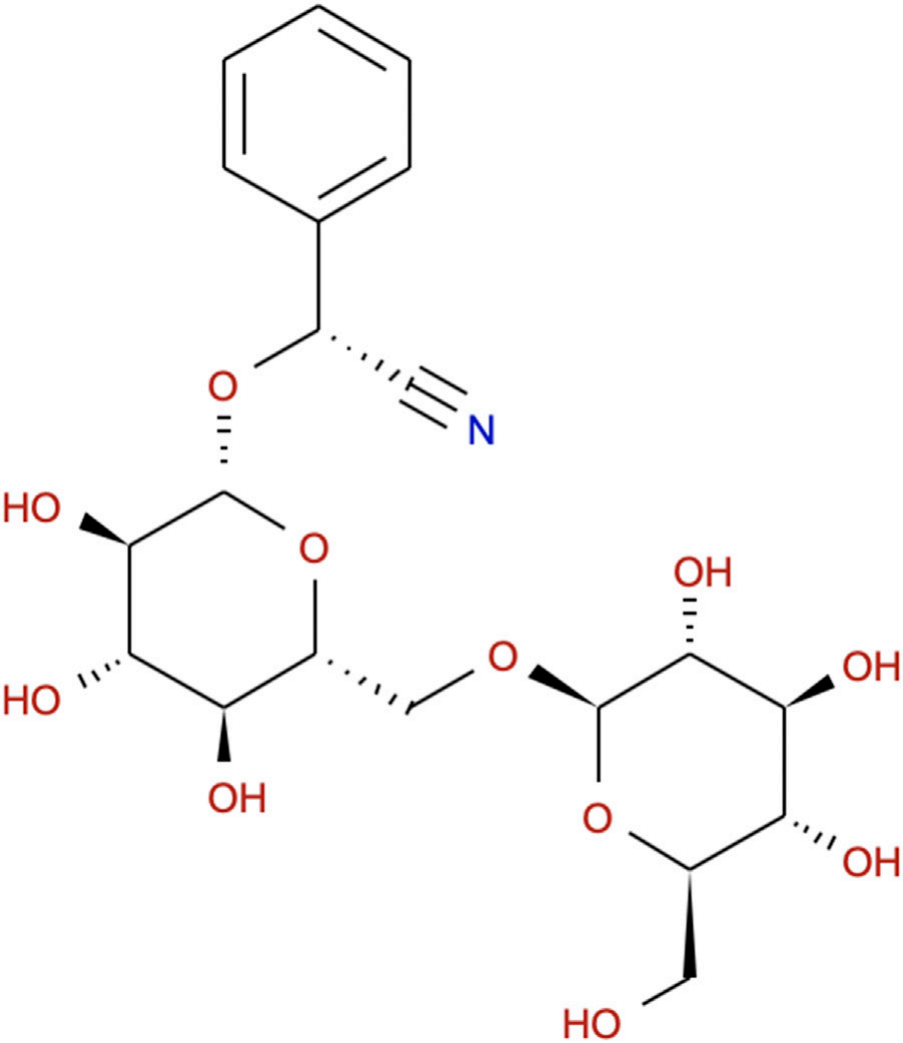
Chemical structure of AMY.

In recent years, an increasing number of studies have been carried out to explore the therapeutic potentials of natural medicinal ingredients (Chung et al., 2010; Patil et al., 2019), due to their safety, effectiveness, fewer side effects and economic efficiency (Uddin et al., 2014; Scheiman 2016). Extracts from raw herbal drugs usually have multiple components, which can induce various biological activities. Some extracts are effective in curing inflammatory ailments and atherosclerosis (Bai et al., 2019; Wang et al., 2019). Therefore, they could be desirable substitutes for synthetic anti-inflammatory compounds. Prunus persica (L.) Stokes (PP) has been used to treat arthritis for its effect to curb inflammation (Lee et al., 2016) and activate blood circulation (Liu et al., 2012). Amygdalin (AMY, molecular formula: C_20_H_27_NO_11_, Figure 1), the main component of PP, could suppress the production of prostaglandin E (2) synthesis, nitric oxide synthase 1 (Nos1), Il-17A, Il-23 and p- Mapk14 protein *in vitro* (Yang et al., 2007; Zhong et al., 2018). *In vivo*, AMY exerts a protective effect against inflammation-related disease associated with decreased Tnfα, Il-1, Il-6, soluble intercellular adhesion molecule-1 and NF-κB (Luo et al., 2015; Zhang et al., 2017). The anti-atherosclerosis and lipid-lowering function of AMY has been proved to be closely associated with suppression of Toll-like receptors in atherosclerotic mice (Jiagang et al., 2011; Lv et al., 2017; Zhao and Yang, 2012). However, the anti-inflammatory and anti-atherosclerotic mechanism of AMY is still unclear. Especially, whether AMY inhibits the proliferation of heart lymphatic vessels to control the inflammation in atherosclerosis stays unknown.

The aim of this study was to investigate the curative effect of AMY on atherosclerosis and its anti-inflammatory role in ApoE^−/−^ mice and BMDMs, particularly its association with mitogen-activated protein kinases (MAPKs), activator protein-1 (AP-1) and nuclear factor-kappa B (NF-κB) p65 signaling pathways.

## Methods

### Reagent

AMY was extracted from PP (purity: 99.28%, available at: http://www.cdmust.com) by Mansite Biological Company (Chengdu, CN). High-fat diet (HFD, 78.85% normal diet +21% lard +0.15% cholesterol) was bought from SYSE Biotechnology Co., Ltd. (Changzhou, CN). oxLDL (20605ES05) was purchased from Yeasen Biotech Co., Ltd. (Shanghai, Chian). 4′,6-diamindino-2-phenylindole (DAPI, P0131), Triton X-100 (P0096), BCA protein assay kit (P0012), interleukine (Il)-6, Il-10, tumor necrosis factor (Tnf) *α* enzyme-linked immunosorbent assay (ELISA, P1326) and Enhanced Cell Counting Kit-8 (CCK-8, C0042) were purchased from Beyotime Biotechnology (Shanghai, CN). Nos1 Assay Kit was purchased from Jiancheng Bioengineering Institution (Nanjing, CN). PrimeScript RT Reagent Kit for RT-PCR amplification (# RR037A) and TB Green Premix EX Taq (# RR420A) were purchased from TAKARA Biomedical Technology Co., Ltd. (Beijing, CN). RNA Purification Kit was purchased from EZBioscience (Shanghai, CN). Antibodies against: Mapk1 (# 4,695, 1:500), phospho- Mapk1 (# 4,370, 1:500), Mapk8 (# 9,258, 1:500), phospho- Mapk8 (# 4,668, 1:500), Mapk14 (# 8,690, 1:500), phospho- Mapk14 (# 4,511, 1:500), monoclonal NF-κB (# 8,242, 1:400), Tnfα (# 11,948, 1:200), Gapdh (# 5,174, 1:5,000), goat anti-rabbit IgG H&L (Alexa Fluor^®^ 647, # 4,414, 1:1,000) and goat anti-rabbit IgG H&L (Alexa Fluor^®^ 488, # 4,412, 1:1,000) were obtained from Cell Signaling Technology Inc. (Beverly, Massachusetts, USA); lymphatic vessel endothelial hyaluronan receptor 1 (Lyve-1, ab14917, 1:100), nitric oxide synthase 2 (Nos2, ab153223, 1:1,000) was purchased from Abcam (Cambridge, United Kingdom). Primers for Il-6, Il-10, Tnfα, Nos2, and Actb were from Sangon Biotech Co., Ltd. (Shanghai, CN). Mapk14 activator (asiatic acid, S2266), Mapk8 activator (anisomycin, S7409) and Mapk1 activator (LM22B-10, S6760) were purchased from Sellect (Shanghai, CN). Dulbecco’s Modified Eagle Medium (DMEM) high glucose medium and fetal bovine serum (FBS) were purchased from Fisher Scientific International Inc. (PIT, USA).

### Animals

The animal study was approved by the Ethics Committee of Longhua Hospital Affiliated to Shanghai University of Traditional Chinese Medicine (No.: 2019-N002, shown in supplementary information). Male ApoE^−/−^ mice were purchased from GemPharmatech Co., Ltd. (Nanjing, Jiangsu, http://www.gempharmatech.com). All the mice were bred at the Animal Center of Longhua Hospital Affiliated to Shanghai University of Traditional Chinese Medicine.

We conducted a pre-experiment to explore the AMY concentration for injection. Then, 40 ApoE^−/−^ mice were randomly divided into four groups (10 mice/group): model group (MOD), high AMY (0.08 mg/kg) group (AH), low AMY (0.04 mg/kg) group (AL), and simvastatin (2.57 mg/kg) group (ST). AMY, which was in the form of water soluble white powder, was resolved in 0.9% saline. Then we use specific degerming filters with holes of 0.22 μm diameter to filtrate the AMY solution. After eight weeks’ high-fat diet (HFD) feeding, the mice were intraperitoneally injected with AMY or received oral simvastatin for 12 weeks daily. MOD mice was injected with the same volume of 0.9% saline. During the injection period, mice were also fed with HFD. Additionally, 10 ApoE^−/−^ mice, as control group (CON), were fed with normal diet and injected with the same volume of 0.9% saline daily. All the mice aged between 6 and 8 weeks. The body weight of the mice was measured once a week during the experiment. The mice were fasted 12 h before sacrifice via carbon dioxide asphyxiation.

### Cell Culture

Bone marrow-derived cells were extracted from the femur and tibia of 6-week-old ApoE^−/−^ mice. Macrophage colony stimulating factor was used to induce the bone marrow-derived cells to form BMDMs. The cells grown in DEME high glucose medium with 10% FBS were maintained at 37°C in a humidified 5% CO_2_ incubator. For the activators (Sellect, CN), BMDMs were treated with 10 μM Mapk1 activator, Mapk8 activator or Mapk14 activator for 0.5 h, and then with 20 μg/ml oxLDL for another 23.5 h.

The oxLDL was prepared as follows: human low density lipoprotein was oxidized in phosphate buffer saline containing 10 μM Cu_2_SO_4_, and excessive ethylene diamine tetraacetic acid was added to stop the oxidation. The purity of the oxLDL was higher than 97% and concentration was 1.0–3.9 mg/ml.

### Cell Viability Assay

BMDMs (5 × 10^4^ cells/well) were seeded into 96-well plates and incubated with various concentrations of AMY (25, 50, 100, 200, 400, and 800 μg/ml) for 24 h. Cells were added with CCK-8 (Beyotime Biotechnology, CN) solution (10 μL/well) and incubated for 1 h. Then the absorbance of each well was measured under 450 nm to determine cell viability.

### Real-Time Quantitative PCR

To analyze gene expressions of Il-6, Il-10, Tnfα and Nos2, BMDMs were pre-protected with AMY for 0.5 h and then stimulated with oxLDL for 23.5 h. Total RNA was extracted using RNA Purification Kit (EZBioscience, CN) according to the manufacturer's instructions. Reverse transcription was performed using 1 μg RNA to synthesize cDNA with PrimeScript RT Reagent Kit (TAKARA, CN) into the 20 μL-volume system according to the manufacturer’s instructions. Then 60 μL double distilled water was added to dilute to 80 μL. In ApoE^−/−^ mice, aortic mRNA was extracted and reversed in the same way.

For Real-time quantitative PCR (RT-PCR) using TB Green Premix EX Taq with fluorescence quantitative PCR instrument (Applied Biosystems), 4 μL of total RNA was incubated with 1 μL of primer (0.1 µg/µL), 10 μL of TB Green, 0.4 μL of ROXⅡ enzyme and 4.6 µL of double distilled water. PCR primers were designed using online website PrimerBLAST of National Center for Biotechnology Information and synthesized by Sangon Biotech Co., Ltd. (Shanghai, CN). Primer sequences in this experiment were shown in Table 1.

**TABLE 1:** List of primers for real-time PCR analysis.

GY	AN	LA	SS	LE	PL	SV	PS	EA (%)	FC (μmol/L)
Actb	NM_007393.5	F: 30–46 R: 197–261	Primer-BLAST	1, 2	187	None	F: 5′- ACT​GTC​GAG​TCG​CGT​CC-3′	94.968	0.25
							R: 5′- CCC​ACG​ATG​GAG​GGG​AAT​AC-3′		
Il-6	NM_001314054.1	F: 115–134 R: 539–561	Primer-BLAST	1, 4	447	Variant 2	F: 5′- ACT​GTC​GAG​TCG​CGT​CC-3′	95.344	0.25
							R: 5′- CCC​ACG​ATG​GAG​GGG​AAT​AC-3′		
Il-10	NM_010548.2	F: 482–501 R: 709–729	Primer-BLAST	1, 2	248	None	F: 5′-GTG​GAG​CAG​GTG​AAG​AGT​GA-3′	108.473	0.25
							R: 5′-TCG​GAG​AGA​GGT​ACA​AAC​GAG-3′		
Tnfα	NM_013693.3	F: 211–230 R: 399–422	Primer-BLAST	4	212	Variant 1	F: 5′-AGG​CAC​TCC​CCC​AAA​AGA​TG-3′	98.08	0.25
							R: 5′-TTG​AGA​AGA​TGA​TCT​GAG​TGT​GAG-3′		
Nos2	NM_010927.4	F: 122–143 R: 225–243	Primer-BLAST	1, 7	122	Variant 1	5′- GTG​AAG​GGA​CTG​AGC​TGT​TAG​A-3′	92.417	0.25
							5′- GCA​CTT​CTG​CTC​CAA​ATC​C-3′		

Notes: GY, gene symbol; AN, accession number; LA, location of amplicon; SS, specificity screen; LE, location of each primer by exon; PL, product length; SV, slice variants; PS, primer sequence; EA, efficiency of amplification; FC, final concentration.

### Measurement of Nitric Oxide Synthase 1

Nos1 was measured using the Assay Kit (Jiancheng Bioengineering Institution, CN). Cells were planted at 4 × 10^5^ cells/well in 96-well plates, pre-protected with various concentrations of AMY for 0.5 h, and then incubated with 20 µg/ml oxLDL for 23.5 h. Cell-free supernatant was used to detect Nos1 production and finally determined at 540 nm using a microplate reader (BIO-TEK).

### Assessment of Serum Lipid in the ApoE^−/−^ Mice

Blood samples collected under chloral hydrate anesthesia were kept still for 1 h at room temperature and then centrifuged for 15 min at 18 × g. The upper transparent serum was transferred into a new tube and diluted with double distilled H_2_O (2:3) for examinations. Total cholesterol (CHOL), triglyceride (TG), high density lipoprotein cholesterol (HDL) and low density lipoprotein cholesterol (LDL) levels were detected as previously described (Wang et al., 2020).

### ELISA for Quantitative Analysis of Il-6, Il-10 and Tnf**α**

Il-6, Il-10 and Tnfα (pg/ml) in the culture medium was determined using an ELISA kit (Beyotime, CN) according to the manufacturer’s instructions. In ApoE^−/−^ mice, the serum protein expression of these cytokines was detected by ELISA. In addition, BMDMs (4 × 10^5^ cells/well) in 96-well plates were treated with various concentrations of AMY for 0.5 h, followed by incubation with 20 µg/ml oxLDL for 23.5 h. Mouse blood serum was obtained as described before.

### Western Blot Assays of Nos2, MAPKs and AP-1

To detect Nos2 protein expression, BMDMs (6 × 10^5^ cells/well) were seeded into 6-well plates, pre-treated with different doses of AMY for 0.5 h, and then with oxLDL for 23.5 h.

To reveal the MAPKs (Mapk1, Mapk14 and Mapk8) and AP-1 (Fos and Jun) pathways, cells were pre-treated with AMY for 30 min and then with oxLDL for 30 min since phosphorylated proteins levels of BMDMs could peak within 30 min (Wei et al., 2016). After treatment, cells were washed twice with phosphate buffer saline, added with RIPA (radio immunoprecipitation assay) Lysis buffer, then collected into 1.5 ml microcentrifuge tubes to fully dissociate for 30 min, and shaken completely every 10 min on a vortex machine. The whole protein was extracted from the supernatant after centrifugation. Protein contents were measured with the BCA protein assay kit (Beyotime, CN). The proteins were separated with 10% sodium dodecyl sulfate polyacrylamide gel electrophoresis for 80 min and transferred onto a polyvinylidene fluoride membrane for 50 min. After being blocked with 2.5% BSA for 1 h, the membrane was incubated in a sealed plastic box with each primary antibody diluted with 2.5% BSA overnight at 4°C. Subsequently, the membrane was washed using Tris-buffered saline with Tris-buffered saline with Tween-20 (TBST) and incubated with the appropriate secondary antibody for 1 h. After washing the membrane with TBST for five times for 50 min, the signal was visualized using an electrochemiluminescence (ECL) western blot kit (Beyotime, CN). The bands were visualized and photographed using the ChemiScope 6000 imaging machine (CLINX, CN). MAPKs of mice aorta were detected in the same way.

### Oil Red O Staining for Mouse Aortic Sinus Lipid-Rich Plaque

Frozen slices were made as follows: the fresh tissues were fixed in 4% paraformaldehyde at 4°C for 24 h, then dehydrated in 10, 20, and 30% sucrose solutions for 24 h, respectively, and finally embedded with OCT glue to make frozen slices. The heart was cross-sectioned into 10 µm-thickness to reveal the atrium and aortic sinus. Frozen slices were stained with Oil Red O solution (0.5% in isopropanol, diluted with double distilled water in ratio of 3:2) for 1 h at room temperature and counterstained with hematoxylin for 2 min.

### Immunofluorescence Staining

BMDMs (4 × 10^5^ cells/well) were seeded into 12-well plates and incubated with or without oxLDL or AMY. After a 24 h-incubation, cells were washed twice with phosphate buffer saline, then fixed with formaldehyde and permeabilized by 0.2% Triton X-100. The antibody was added to each slide, shaken slightly on a shaker overnight at 4°C. Then, the slides were incubated with anti-rabbit IgG ab and DAPI for 2 h and 2 min, respectively. Finally, cells were observed using a LSM800 confocal fluorescence microscope (ZEISS, Germany). Frozen slices were used to detect lymphatic vessels of heart atrium in the same way.

### Statistical Analysis

Graph Pad Prism software (version 8.4.0) was used to analyze the results. Western Blot results were measured by ImageJ software. The results were shown as the mean ± standard deviation (SD). One-way ANOVA was used to determine statistically significant differences among groups by Dunnett post-hoc analysis. Statistically significant difference was determined as *p* < 0.05.

## Results

### AMY Lowered Body Weight and Improved Serum Lipid Profiles in Mice

**FIGURE 2:**
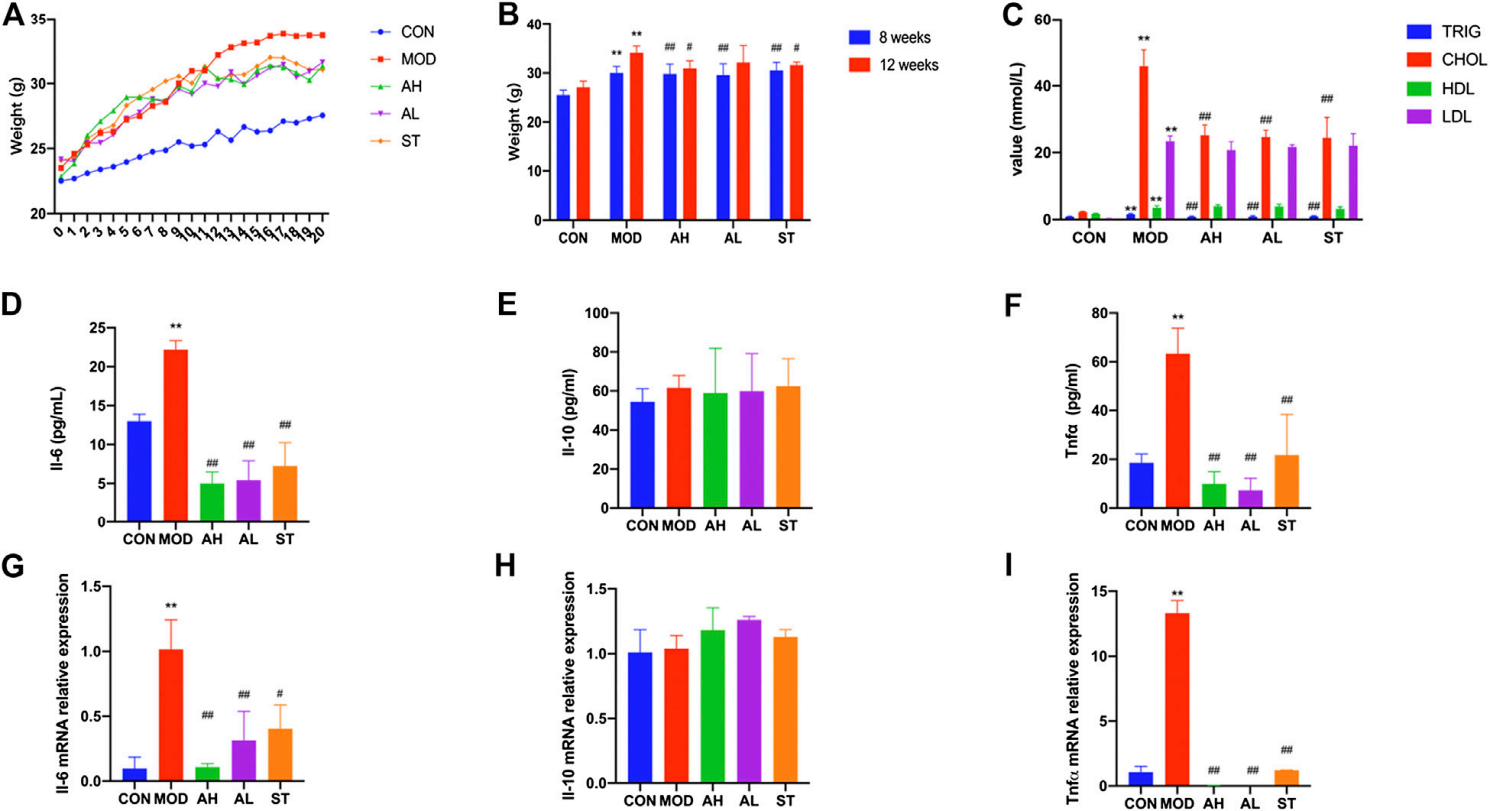
Body weight, serum lipids and inflammation cytokines of mice. Body weights of ApoE^−/−^ mice are shown **(A,B)**. Week 0–1: 1 week’s adaption to environment; Week 1–8: HFD induction; Week 9–20: intervention. TG, CHOL, HLD and LDL levels of serum were detected **(C)**. Production of Il-6, Il-10 and Tnfα in plasma was measured by ELISA kit **(D–F)**. Aorta gene expression of Il-6, Il-10 and Tnfα was detected by RT-PCR method **(G–I)**. CON: control group (normal diet); MOD: model group (HFD); AH: high AMY group (HFD plus 0.08 mg/kg AMY); AL: low AMY group (HFD plus 0.04 mg/kg AMY); ST: simvastatin group (HFD plus 2.57 mg/kg simvastatin). TG, triglyceride; CHOL, total cholesterol; HDL, high density lipoprotein cholesterol; LDL, low density lipoprotein cholesterol. MOD group was compared to CON group. AMY or ST group was compared to MOD group. Data are expressed as mean ± SD. *p < 0.05, **p < 0.01 vs. CON group; #*p* < 0.05, ##*p* < 0.01 vs. MOD group.

The mice in MOD, AH, AL and ST groups had much higher body weights than those in CON group at Week 8 (*p* < 0.01). The body weight of mice in AH group was lower than those in MOD group after 12 weeks’ intervention (*p* < 0.01, Figures 2A,B).

Serum samples were collected to measure levels of TG, CHOL, HDL and LDL. As shown in Figure 2C, all the four indexes in MOD group were higher than those in CON group (*p* < 0.01). Compared to the MOD group, TG and CHOL levels in AH, AL and ST groups were lower (*p* < 0.01). But no significant differences in HDL and LDL levels were found.

### AMY Attenuated Inflammatory Cytokine Expression in Mice

We used ELISA method to detect three typical inflammatory cytokines: Il-6, Il-10 and Tnfα. As shown in Figures 2G–I, the plasma levels of Il-6 and Tnfα in MOD group increased compared with those in CON group (*p* < 0.01, *p* < 0.01), but significantly decreased after 12 weeks’ AMY and ST treatment compared to those in MOD group before the treatment (*p* < 0.01). No significant difference in Il-10 expression was found among these groups.

The results were consistent with the changes in Tnfα mRNA level of mice aorta analyzed by RT-PCR (*p* < 0.01). Il-6 mRNA level increased in MOD group and decreased in AH and AL group (*p* < 0.05). Il-10 mRNA level in MOD group was lower than that in CON group, but higher in ST group than that in MOD group (*p* < 0.01) (Figures 2D–F).

### AMY Decreased Atherosclerotic Lesion Area of Aortic Sinus and Lymphatic Vessels in Heart Atrium

**FIGURE 3:**
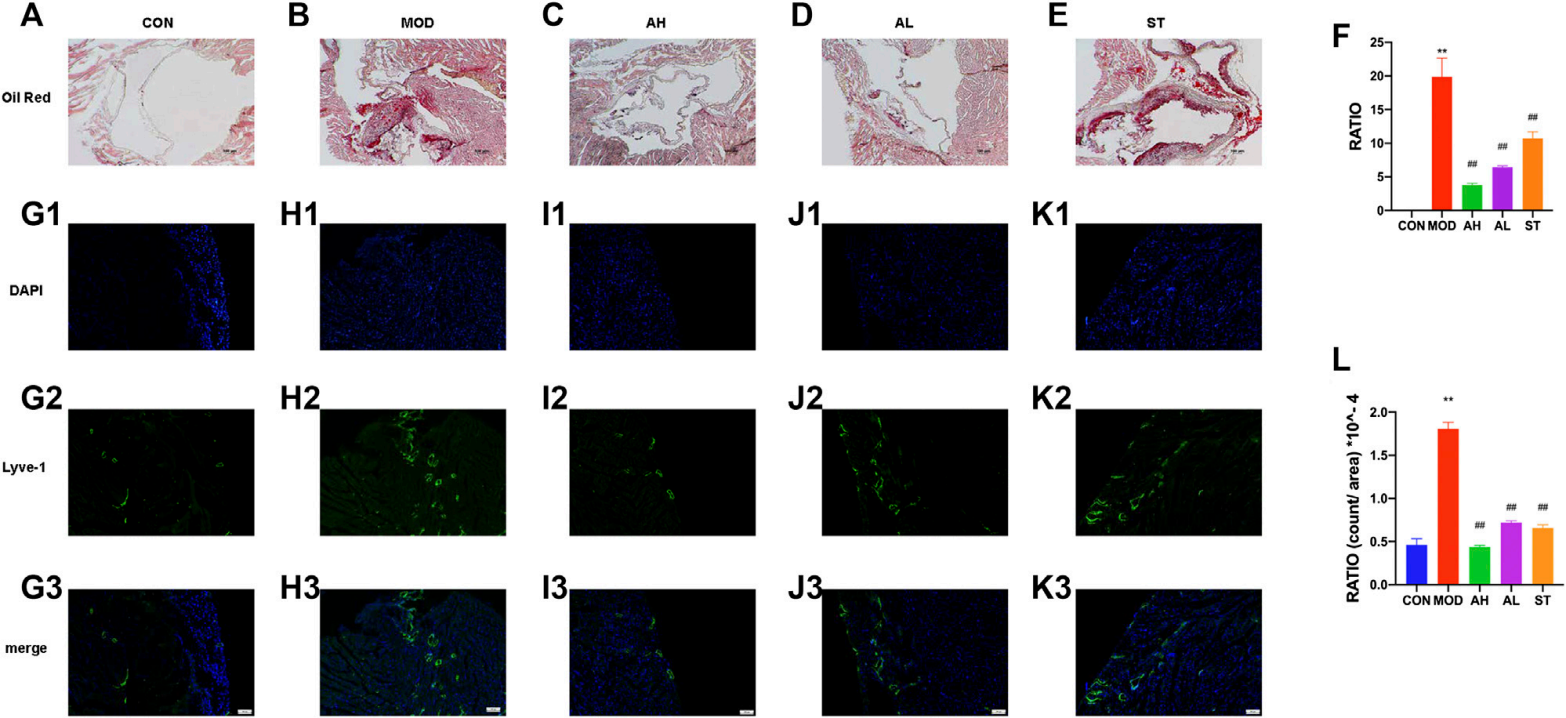
Atherosclerotic plague in the aortic sinus and lymphatic vessels in heart atrium. Oil Red O staining was used to detect the progress of lipid-rich plaques in CON **(A)**, MOD **(B)**, AH **(C)**, AL **(D)** and ST group **(E)**. A quantitative analysis was shown in **(F)**. The ratio refers to the relative area of atherosclerotic plague to that of aortic sinus. Immunofluorescence staining method was used to show the proliferation of lymphatic vessels among CON **(G1–G3)**, MOD **(H1–H3)**, AH group **(I1–I3)**, AL group **(J1–J3)** and ST group **(K1–K3)**. A quantitative analysis was shown in **(L)**. CON, control group; MOD, model group; AH, high AMY group; AL, low AMY group; ST, simvastatin group. MOD group was compared to CON group. AMY or ST group was compared to MOD group. Data are expressed as mean ± SD. **p < 0.01 vs. CON group; ##*p* < 0.01 vs. MOD group.

Lipid-rich plaques areas were assessed by Oil Red O staining. As shown in Figures 3A,B, lipid-rich plaque was not detected in CON group, but clearly observed in ApoE^−/−^ AS mouse model. In contrast, significant reduced plaque area was detected in AMY and ST groups (Figures 3C–E). The quantitative analysis was shown in Figure 3F.

Lyve-1 expression of epicardium in heart atrium was assessed to examine lymphatic vessel proliferation by immunofluorescence staining. As shown in Figures 3G1–H3, the lymphatic vessels in MOD group increased and were discontinuous compared with those in CON group. However, the lymphatic vessels in AMY and ST groups were less than the MOD group (Figures 3I–K3). The quantitative analysis was shown in Figure 3L.

### Effects of AMY on BMDMs Viability

**FIGURE 4:**
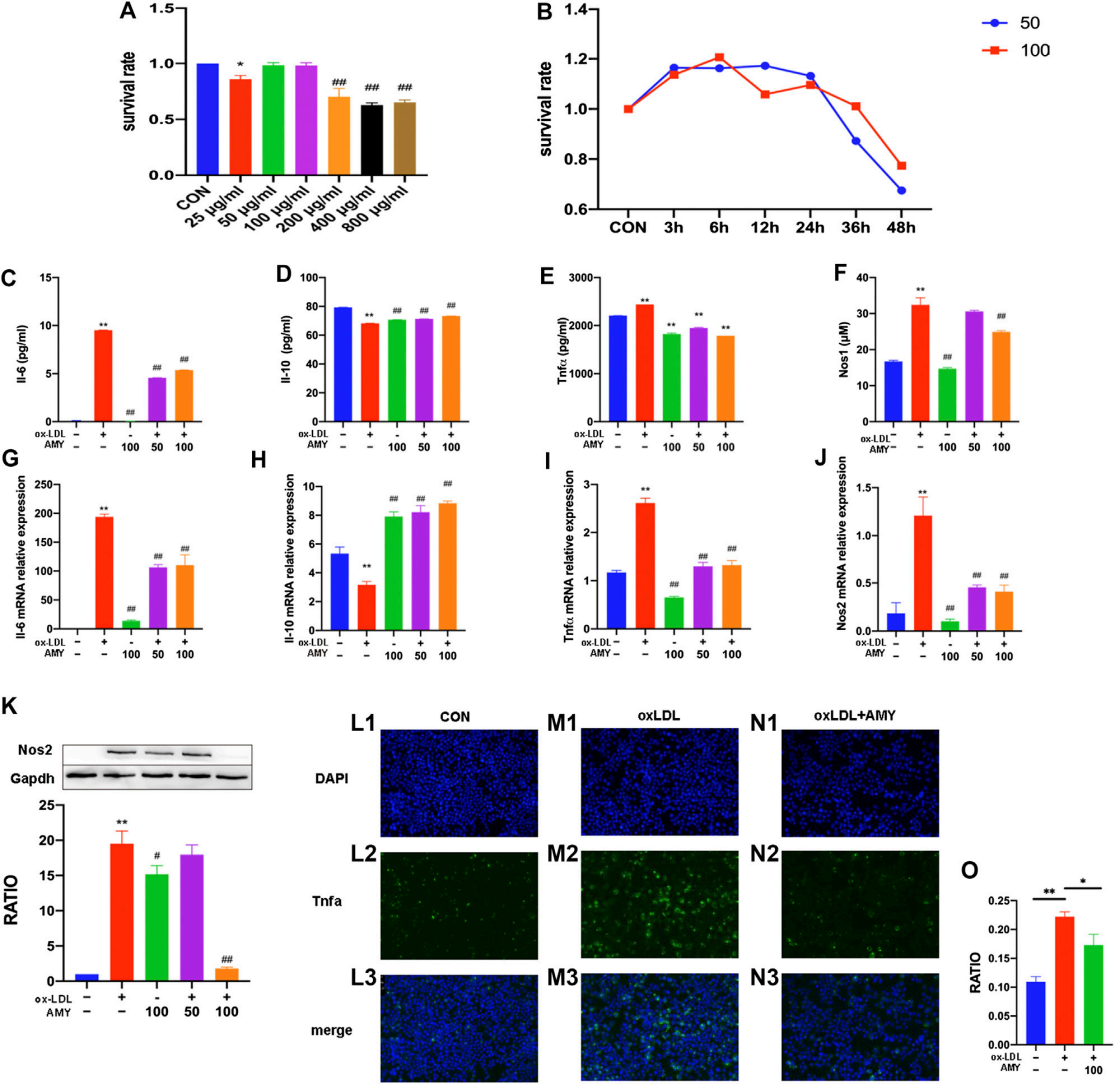
Il-6, Il-10, Tnfα, Nos2 and Nos1 production in BMDMs. Dose-effect **(A)** and time-effect **(B)** of AMY on viability of BMDMs. Total RNA was extracted, and then gene levels of Il-6 **(C)**, Il-10 **(D)**, Tnfα **(E)**, and Nos2 **(F)** were detected by PCR. Il-6 **(G)**, Il-10 **(H)** and Tnfα **(I)** were evaluated by ELISA kit. Nos1 production was detected by Nos1 kit **(J)** and Nos2 was explored by WB **(K)**. Moreover, Tnfα was shown by immunofluorescence staining among CON **(L1–L3)**, oxLDL **(M1–M3)** and oxLDL+ AMY **(N1–N3)** groups. The quantitative analysis is shown in **(O)**. The ratio refers to the relative number of positive cells to the total number of cells. Data are expressed as mean ± SD. **p < 0.01 vs. group without ox-LDL and AMY; #*p* < 0.05, ##*p* < 0.01 vs. group with ox-LDL only.

AMY did not bring any significant cytotoxicity to BMDMs at concentrations of 50 and 100 µg/ml within 24 h. Therefore, these two concentrations were adopted in the subsequent cell experiments (Figures 4A,B).

### AMY Attenuated Inflammatory Cytokine (Il-6, Il-10 and Tnf**α**), **Nos1 and Nos2 Expressions in BMDMs**

After 0.5 h AMY-pretreatment, BMDMs were stimulated with oxLDL for 23.5 h. As shown in Figures 4C–F, oxLDL alone significantly increased the mRNA levels of Il-6, Tnfα and Nos2 and decreased mRNA level of Il-10 (*p* < 0.01). AMY at 50 and 100 µg/ml decreased the oxLDL-induced elevation in mRNA levels of Il-6, Tnfα and Nos2 and reversed the decreased mRNA level of Il-10 (*p* < 0.01).

As shown in Figures 4G–K, oxLDL increased the production of Il-6, Tnfα, Nos1 and Nos2 and decreased Il-10 production (*p* < 0.01). Only 100 µg/ml AMY decreased Il-6, Nos1 and Nos2 (*p* < 0.01). AMY at both 50 and 100 µg/ml decreased Tnfα and increased Il-10 production (*p* < 0.01).

We also used immunofluorescence method to detect Tnfα expression (Figures 4L1–N3) and perform quantitative analysis (Figure 4O). The oxLDL exposure markedly increased Tnfα generation compared to control cells. However, AMY treatment (100 μg/ml) suppressed the oxLDL-induced Tnfα production.

### AMY Decreased MAPKs/AP-1/NF-κB in Mouse Aorta and BMDMs

**FIGURE 5:**
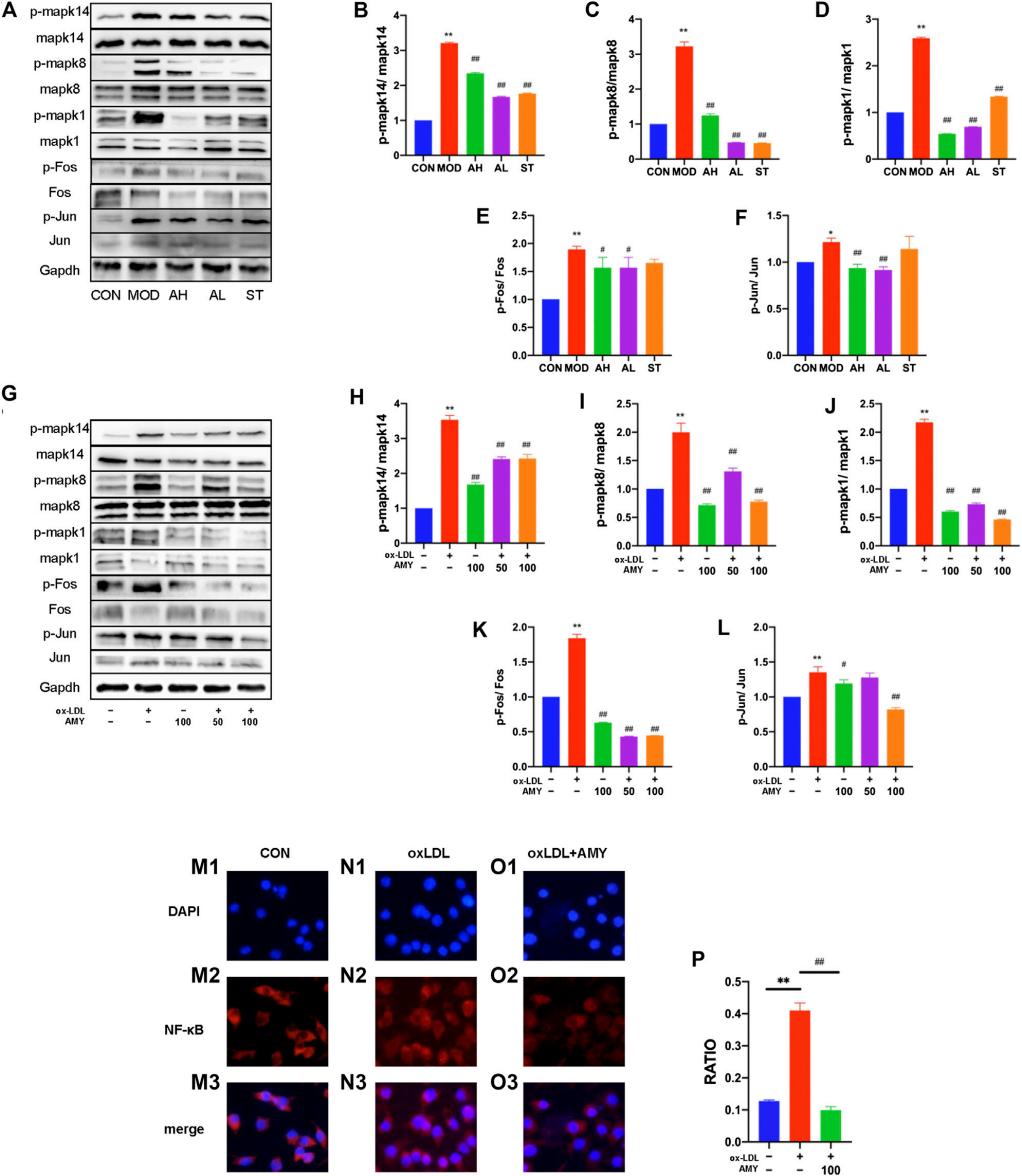
AMY on MAPKs/AP-1/NF-κB p65 signaling pathway in mouse aorta and BMDMs. **(A)** Total and phosphorylated levels of Mapk14 **(B)**, Mapk8 **(C)**, Mapk1 **(D)**, Fos **(E)**, and Jun **(F)** in mouse aorta were determined by WB method. **(G)** Total and phosphorylated levels of Mapk14 **(H)**, Mapk8 **(I)**, Mapk1 **(J)**, Fos **(K)**, and Jun **(L)** in BMDMs were determined by WB analysis. NF-κB p65 expression was determined by measuring more than 200 cells from CON **(M1–M3)**, oxLDL **(N1–N3)** and oxLDL+ AMY **(O1–O3)** groups using immunofluorescence staining method. The quantitative analysis was shown in **(P)**. CON, control group; MOD, model group; AH, high AMY group; AL, low AMY group; ST, simvastatin group. Data are expressed as mean ± SD. **p < 0.01 vs. CON group, or group without ox-LDL and AMY; #*p* < 0.05, ##*p* < 0.01 vs. MOD group, or group with ox-LDL only.

To testify whether the inflammatory cytokine changes was achieved through MAPKs, phosphorylation of Mapk14, Mapk8 and Mapk1 were examined by WB method. Figures 5A–D showed the significant increase of phosphorylation levels in MOD group, and reduction in AH, AL and ST group (*p* < 0.01).

AMY at 50 and 100 μg/ml was employed to treat oxLDL-induced cells, and phosphorylation of Mapk14, Mapk8, and Mapk1 was examined by WB assays. The results showed oxLDL significantly stimulated phosphorylation of MAPKs in BMDMs, but a significant reduction was found in the phosphorylation of these kinases under AMY intervention (Figures 5G–J) (*p* < 0.01).

Considering the relationship between phospho-MAPKs (Mapk14 and Mapk8) and AP-1 activation, we explored the effects of AMY on Fos and Jun with WB assays. Figures 5A,E,F showed the significant increase in *p*-Fos and *p*-Jun levels in MOD group, and reduction in AH and AL group (*p* < 0.05, *p* < 0.01) As shown in Figures 5G,K,L, AMY treatment significantly inhibited the phosphorylation of both Fos (at 50 and 100 μg/ml) (*p* < 0.01) and Jun (only at 100 μg/ml) (*p* < 0.01).

Furthermore, the effects of AMY on NF-κB p65 nuclear translocation were investigated using immunofluorescence staining. The results clearly showed that oxLDL stimulation led to a decrease of NF-κB p65 in cytoplasm and an increase of NF-κB p65 in nucleus, indicating that NF-κB p65 had been translocated to the nucleus following oxLDL stimulation. However, AMY (100 μg/ml) treatment decreased the oxLDL-induced NF-κB p65 levels in the nucleus (*p* < 0.01) (Figures 5L–O).

### AMY Decreased Inflammatory Cytokine Levels Mainly Through the MAPKs Signaling Pathway

**FIGURE 6:**
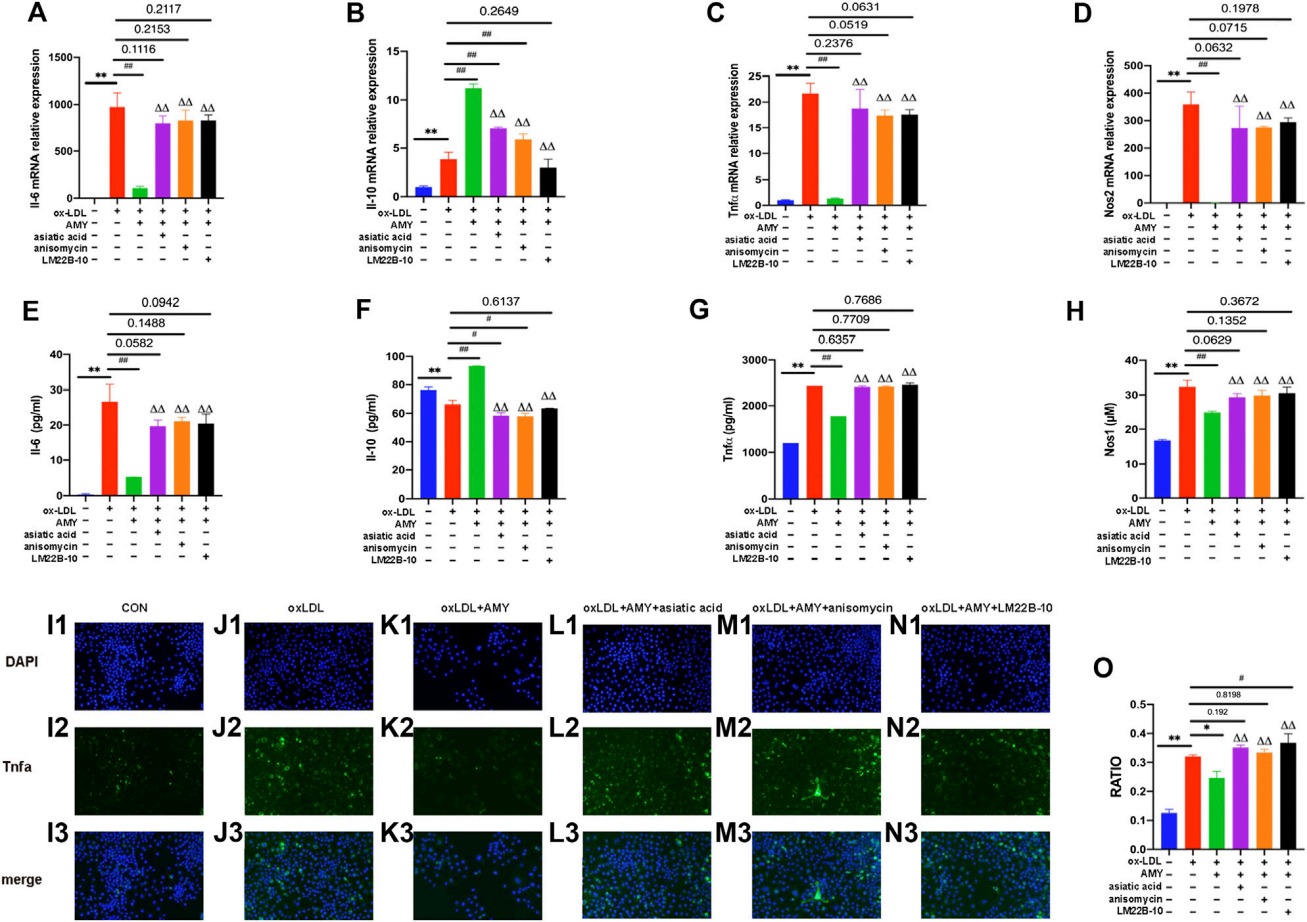
AMY functions mainly through the MAPKs signaling pathway. Cells were pretreated with 10 μM Mapk14 activator (asiatic acid), 10 μM Mapk8 activator (anisomycin), or 10 μM Mapk1 activator (LM22B-10) for 0.5 h, then treated with oxLDL for 23.5 h. Total RNA was extracted and then gene levels of Il-6 **(A)**, Il-10 **(B)**, Tnfα **(C)** and Nos2 **(D)** were detected by PCR. Il-6 **(E)**, Il-10 **(F)**, Tnfα **(G)**, and Nos1 **(H)** were evaluated by ELISA or Nos1 kit. Tnfα in CON **(I1–I3)**, oxLDL **(J1–J3)**, oxLDL+ AMY **(K1–K3)**, oxLDL+ AMY+ Mapk14 activator **(L1–L3)**, oxLDL+ AMY+ Mapk8 activator **(M1–M3)** and oxLDL+ AMY+ Mapk1 activator **(N1–N3)** groups was detected by immunofluorescence staining. The quantitative analysis is shown in **(O)**. The ratio means the relative number of positive cells to the number of all cells. Data are expressed as mean ± SD. **p < 0.01 vs. group without ox-LDL and AMY; #*p* < 0.05, ##*p* < 0.01 vs. group with ox-LDL only; ΔΔ *p* < 0.01 vs. group with ox-LDL and AMY.

To further verify the role of MAPKs in the anti-inflammatory function of AMY, three specific activators were implied. As shown in Figures 6A–D, gene expressions of Il-6, Tnfα and Nos2 were reduced and Il-10 was increased after AMY treatment compared to the oxLDL-induced group (*p* < 0.01). There is no significant difference between activators-treated groups and oxLDL-induced group. Figures 6E–H showed that AMY reduced oxLDL-induced Il-6, Tnfα and Nos1 production (*p* < 0.01), and augmented Il-10 expression (*p* < 0.01). There is no difference between activator-treated groups and oxLDL-induced group except for the Il-10 production with anisomycin and LM22B-10. Immunofluorescence pictures showed Tnfα expression was increased in activator-treated BMDMs with AMY and oxLDL, but reduced in oxLDL and AMY-treated groups (Figure 6I1–O).

## Discussion

Inflammation is a biological response to eliminate harmful incentives, such as infection or harmful irritants (Khatami 2008). Macrophages, the most important antigen-presenting cells (APCs) of innate immunity (Wei et al., 2016), play a central role in inflammation and host defense. Chronic inflammation can activate various cytokines to aggravate diseases (Patil et al., 2019; Pelletier-Galarneau and Ruddy, 2019). Macrophage polarization is involved in the plaque formation of atherosclerosis (Shapouri-Moghaddam et al., 2018). Expression markers of all the subtype macrophages are CD68, F4/80, CD64 and MHC Ⅱ (Klopfleisch, 2016). Effector molecules of M1 macrophage include Il-6, Tnfα, and Il-12; all subtype M2 macrophages express Il-10 and suppressor of cytokine signaling (Socs) (Klopfleisch, 2016). Il-6 and Tnfα are pro-inflammatory cytokines; but Il-10, a prototypic anti-inflammatory cytokine made primarily by macrophages, can repress the production of matrix metalloproteinase and cyclooxygenase-2 in lipid-loaded macrophage foam cells (Han and Boisvert, 2015). Because of their versatility, macrophages have been primarily targeted in new therapies for cardiovascular diseases. One strategy is to block pro-inflammatory cytokines released by macrophages (Barrett, 2020; Nasser et al., 2020). Therefore, regulating these inflammatory cytokines may attenuate atherosclerosis. Our experiment demonstrated a simultaneous reduction in body weight, serum lipids, plaque area and inflammation cytokines in AMY-treated ApoE^−/−^ mice. Additionally, in oxLDL-stimulated BMDMs, AMY effectively increased anti-inflammatory factor Il-10 and suppressed pro-inflammatory factor Il-6, Tnfα, Nos1 and Nos2.

Lymphatic vessels proliferate in the heart and plaques in CAD and progressive atherosclerosis (Kholova et al., 2011; Telinius and Hjortdal, 2019), a mechanism that could be used to design a new treatment strategy (Vuorio et al., 2017). Previous studies also found that some herbs could decrese the proliferation of lymphatic vessel (Wang Y, et al.). Our results were in accordance with the previous reports: the lymphatic vessels in mouse heart atrium increased in model group, while AMY reversed the proliferation of heart lymphatic vessels in ApoE^−/−^ atherosclerosis mouse model.

Recently, many studies have verified that the performance of many pro-inflammatory cytokines is regulated by MAPKs (Mapk14, Mapk1, and Mapk8) (Jin et al., 2010). We thus investigated the effects of AMY on MAPKs phosphorylation. As the results showed, phospho-MAPKs were decreased after AMY treatment. Using activators, we further testified that AMY inhibited the signaling of MAPKs pathway, which makes it an effective anti-inflammatory agent. After the phosphorylation of Mapk14 and Mapk8, Jun is also phosphorylated to activate AP-1 (Jia et al., 2019). Therefore, we then evaluated the activation of Fos and Jun, revealing that AMY also exerts its anti-inflammatory function through the AP-1 pathway.

NF-κB, an important transcription factor that can positively regulate inflammatory responses, can be activated as oxLDL-TLR binds to the macrophage membrane (Kim et al., 2005). When the activated NF-κB is translocated into the nucleus, the phosphorylated NF-κB can act as a transcription factor of inflammatory genes (Viatour et al., 2005). Our data showed that AMY strongly inhibited oxLDL-stimulated NF-κB p65 translocation, suggesting that AMY can suppress the expression of pro-inflammatory enzymes and cytokines by inhibiting the activity of NF-κB p65.

In this study, the anti-inflammatory mechanism of AMY was investigated. AMY could attenuate atherosclerosis and control the inflammation-related indicators by suppressing the phospho-MAPKs/phospho-AP-1/NF-κB p65 signaling pathway. Additionally, AMY could reverse the proliferation of lymphatic vessels in mouse heart. Taken together, AMY plays a strong anti-inflammatory role in atherosclerosis, and its clinical application is worth further investigation.

## Data Availability Statement

The raw data supporting the conclusions of this manuscript will be made available by the authors, without undue reservation, to any qualified researcher.

## Ethics Statement

The animal study was reviewed and approved by Ethics Committee of Longhua Hospital Affiliated to Shanghai University of Traditional Chinese Medicine

## Author Contributions

PL conceived and designed the experiments. YW and YZ performed the experiments. JW and QJ analyzed the results. YW was a major contributor in writing the manuscript. PL reviewed and edited the final manuscript. All authors read and approved the final manuscript.

## Funding

This work was supported by the National Natural Science Foundation to PL [grant numbers 81873117; 82074200]; the 2018–2020 Three-year Action Plan for Traditional Chinese Medicine Further Development in Shanghai to PL [grant numbers ZY (2018–2020)-CCCX-2002-04].

## Conflict of Interest

The authors declare that the research was conducted in the absence of any commercial or financial relationships that could be construed as a potential conflict of interest.

## GLOSSARY

ApoE^−/−^: ApoE knock-out
oxLDL: oxidized low-density lipoprotein
CAD: coronary artery disease
HSPCs: hematopoietic stem and progenitor cells
BMDMs: bone marrow-derived macrophages
PP: Prunus persica (L.) Stokes
AMY: amygdalin
MAPKs: mitogen-activated protein kinases
AP-1: activator protein-1
NF-κB: nuclear factor-kappa B
HFD: high-fat diet
DAPI: 4′,6-diamindino-2-phenylindole
IL: interleukine
Tnf: tumor necrosis factor
Lyve-1: lymphatic vessel endothelial hyaluronan receptor 1
Nos2: nitric oxide synthase 2
ELISA: enzyme-linked immunosorbent assay
CCK-8: Cell Counting Kit-8
DMEM: Dulbecco’s Modified Eagle Medium
FBS: fetal bovine serum
MOD: model group
AH: high amygdalin group
AL: low amygdalin group
ST: simvastatin group
CON: control group
RT-PCR: Real-time quantitative PCR
Nos1: nitric oxide synthase 1
TBST: Tris-buffered saline with Tween-20
ECL: electrochemiluminescence
SD: standard deviation
TG: triglyceride
CHOL: total cholesterol
HDL: high density lipoprotein cholesterol
LDL: low density lipoprotein cholesterol
APCs: antigen-presenting cells
Socs: suppressor of cytokine signaling
